# Transcriptomic and Metabolomic Analyses Reveal the Importance of Lipid Metabolism and Photosynthesis Regulation in High Salinity Tolerance in Barley (*Hordeum vulgare* L.) Leaves Derived from Mutagenesis Combined with Microspore Culture

**DOI:** 10.3390/ijms242316757

**Published:** 2023-11-25

**Authors:** Hongwei Xu, Nigel G. Halford, Guimei Guo, Zhiwei Chen, Yingbo Li, Longhua Zhou, Chenghong Liu, Rugen Xu

**Affiliations:** 1 Key Laboratory of Plant Functional Genomics of the Ministry of Education/Jiangsu Key Laboratory of Crop Genomics and Molecular Breeding/Jiangsu Co-Innovation Center for Modern Production Technology of Grain Crops/Joint International Research Laboratory of Agriculture and Agri-Product Safety of Ministry of Education of China, Yangzhou University, Yangzhou 225009, China; 2 Shanghai Key Laboratory of Agricultural Genetics and Breeding, Biotechnology Research Institute, Shanghai Academy of Agricultural Sciences, Shanghai 201106, China; 3 Rothamsted Research, Harpenden AL5 2JQ, UK

**Keywords:** barley, mutagenesis, salt tolerance, microspore, transcriptome, metabolome

## Abstract

Barley is the most salt-tolerant cereal crop. However, little attention has been paid to the salt-tolerant doubled haploids of barley derived from mutagenesis combined with isolated microspore culture. In the present study, barley doubled haploid (DH) line 20, which was produced by mutagenesis combined with isolated microspore culture, showed stably and heritably better salt tolerance than the wild type H30 in terms of fresh shoot weight, dry shoot weight, K^+^/Na^+^ ratio and photosynthetic characteristics. Transcriptome and metabolome analyses were performed to compare the changes in gene expression and metabolites between DH20 and H30. A total of 462 differentially expressed genes (DEGs) and 152 differentially accumulated metabolites (DAMs) were identified in DH20 compared to H30 under salt stress. Among the DAMs, fatty acids were the most accumulated in DH20 under salt stress. The integration of transcriptome and metabolome analyses revealed that nine key biomarkers, including two metabolites and seven genes, could distinguish DH20 and H30 when exposed to high salt. The pathways of linoleic acid metabolism, alpha-linolenic acid metabolism, glycerolipid metabolism, photosynthesis, and alanine, aspartate and glutamate metabolism were significantly enriched in DH20 with DEGs and DAMs in response to salt stress. These results suggest that DH20 may enhance resilience by promoting lipid metabolism, maintaining energy metabolism and decreasing amino acids metabolism. The study provided novel insights for the rapid generation of homozygous mutant plants by mutagenesis combined with microspore culture technology and also identified candidate genes and metabolites that may enable the mutant plants to cope with salt stress.

## 1. Introduction

Soil salinity is one of the major abiotic stresses worldwide [1]. With the intensification of climate change, global warming will likely exacerbate the risks of soil salinization. High salt conditions can reduce photosynthetic pigments such as chlorophyll and carotenoids, limit ribulose-1,5-bisphosphatase carboxylase/oxygenase activity and damage the photosynthetic apparatus, leading to poor photosynthesis and a decline in yield [2]. Currently, it is estimated that more than 800 million hectares of agricultural land, including 20% of irrigated land, suffers from soil salinity (https://www.fao.org/soils-portal/soil-management/management-of-some-problem-soils/salt-affected-soils/more-information-on-salt-affected-soils/en/ (accessed on 13 May 2020)) [3,4]. China alone has more than 1.0 × 10^8^ hm^2^ saline soils, which is much more than other countries and regions [5]. Developing highly salt-tolerant varieties is an effective strategy to cope with soil salinization. Therefore, it is extremely urgent for us to rapidly breed highly salt-tolerant varieties, and these would also be good resources to explore salt-tolerance mechanisms. Mutation-based breeding accelerates crop improvement by generating new genetic diversity beyond natural variation. Conventional mutation breeding strategies mainly focus on insertion, and chemical and physical mutagenesis [6]. A major disadvantage of traditional mutation breeding is the subsequent development of homozygous lines, which takes about 6–10 generations of inbreeding by sib-crossing or selfing [7]. The doubled haploid (DH) system offers a number of advantages in plant breeding, as it enables the rapid generation of completely homozygous lines [8], particularly to fix traits and unlock genetic variation [9]. In many species, doubled haploids can be produced via isolated microspore or anther culture [10]. Compared to anther culture, microspores (the immature precursors to pollen grains) are free of anther wall tissues [11]. Furthermore, a remarkable characteristic of microspore cells is their totipotency, which makes them an excellent haploid model to produce DH plants [12]. Therefore, the integration of mutagenesis together with microspore culture technology, which has been reported in many species, such as cabbage (*Brassica oleracea*) [13] and oilseed rape/canola (*Brassica napus*) [14], could provide new opportunities to maximize genetic gains in selection and shorten the breeding cycle. However, there is little information on barley produced by mutagenesis combined with isolated microspore culture, especially regarding the responses to salt stress.

Barley ranks as the fourth most important cereal crop globally and is widely used in food, animal feed and malt production [15]. It is also regarded as being the most salt tolerant among the cereal crops; thus, it is commonly used as a model cereal species for salt-tolerance studies [16]. Genetic research on barley, including for salt-tolerance mechanisms, will be greatly facilitated by the availability of genome sequences [17]. In comparison to the mutants from conventional breeding, the genes of barley mutants after DH technology can be fixed in a homozygous state, and homozygous mutant material is more stable and heritable, providing ideal material for profiling specific gene expression patterns and elucidating the genetics underlying salt tolerance [7].

Numerous studies have been conducted over the past few decades on salt tolerance mechanisms. However, the integrated omics analysis of transcriptomics and metabolomics has provided a new, powerful tool to clarify the relationship of genotype and phenotype. So far, the molecular mechanisms of salinity tolerance in barley genotypes derived from mutagenesis combined with microspore culture have never been elucidated by transcriptomic and metabolomic analyses. Understanding the molecular mechanisms underlying salt tolerance in barley mutants could contribute to the identification of key genes and pathways involved and provide global insight into the characteristics of salinity responses, not only in barley but in cereal species generally. 

The objective of this study was to rapidly generate a barley DH line with superior salt tolerance by mutagenesis combined with isolated microspore culture technology, and explore the mechanisms underlying salt tolerance in the DH line by the integration of transcriptomic and metabolomic profiling.

## 2. Results

### 2.1. Identification of DH20 with Higher Salt Tolerance than H30 by Screening DH Lines Produced by Mutagenesis Combined with Isolated Microspore Culture 

To produce mutagenized lines from barley cv. Hua30, the dry seeds of H30 were treated with Co-60, a mutagen that emits gamma radiation at 500 Gy·min^−1^. After the mutagenesis, seeds were planted in the field and grown to the flowering stage, when spikes were collected and isolated microspore were cultured. Eventually, thirty-one regenerated plants were obtained by natural chromosome doubling and reproduction in Kunming (Yunnan Province). The breeding scheme for the production of a ‘super’ doubled haploid mutant line is shown in Supplement Figure S1.

**Figure 1:**
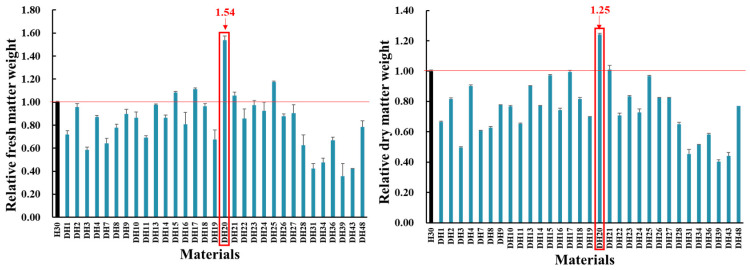
Comparison of relative fresh and dry weight between H30 and mutagenic materials under 360 mM salt concentration at 28 d seedling stage (Zadoks growth scale 19). The relative fresh or dry weight was calculated by comparing the value obtained under salt stress versus control conditions. The relative value for H30 is shown as 1.00, and the relative value for other DH lines is shown as relative to H30. The red line is presented to distinguish the higher relative value for DH lines relative to H30. The red arrows and frame indicate the performance of DH20. All data represent four biological replicates.

As shown in Figure 1, thirty-one DH lines that were produced from H30 via mutagenesis combined with microspore embryogenesis were treated with two levels of salt treatment in hydroponic culture. Among the 31 DH lines, DH20 was identified as showing higher fresh and dry weight than H30 (54% and 25% increase, respectively). 

### 2.2. Comparison of Phenotype and K^+^/Na^+^ Ratio between H30 and DH20 under Control and Salt Conditions

**Figure 2:**
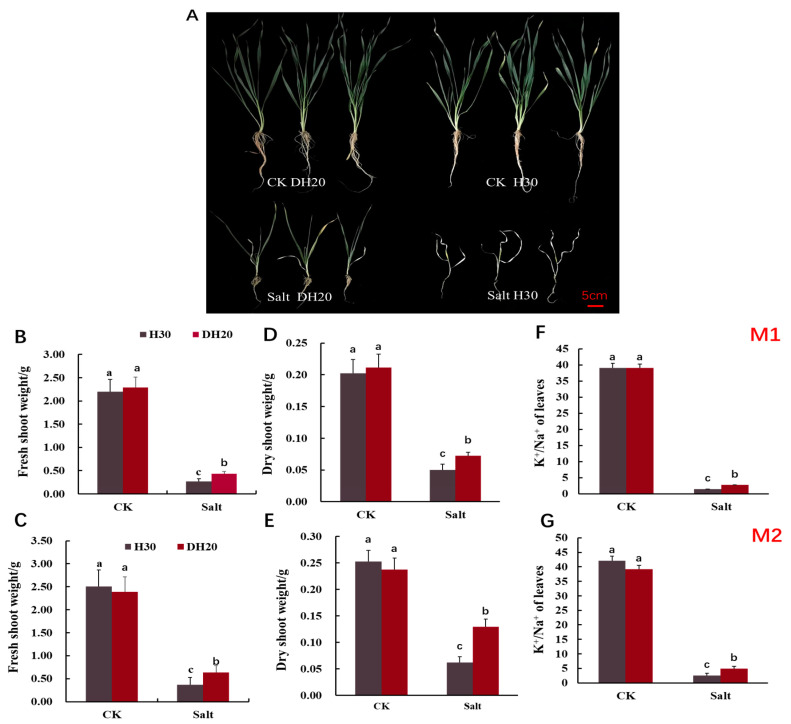
Comparison of H30 and DH20 at 28 d seedling stage (Zadoks growth scale 19) under CK and salt conditions. (**A**) Growth performance; fresh shoot weight in M1 (**B**) and M2 (**C**); dry shoot weight in M1 (**D**) and M2 (**E**); K^+^/Na^+^ of leaves in M1 (**F**) and M2 (**G**). M1 and M2 represents mutagenesis 1 and 2 generation, respectively. Different letters indicate significant differences at the 0.05 level by Student’s *t* test. All data represent three biological replicates. CK: control; Salt: salt condition.

To further evaluate the salt tolerance of DH20, we treated the seedlings with control (CK; 0 mM NaCl) and salt (360 mM NaCl) solutions in hydroponic conditions. No phenotype difference was observed between seedlings of H30 and DH20 under the CK condition. Under the salt treatment, however, the seedlings of H30 exhibited more serious growth inhibition than DH20 (Figure 2A). 

Correspondingly, we further determined the physiological responses of DH20 and H30 exposed to salt stress (Figure 2B–G). No significant differences were detected in the fresh shoot weight, dry shoot weight and K^+^/Na^+^ ratio between H30 and DH20 under normal growth conditions in the M1 and M2 generations. However, DH20 had less of a reduction in fresh shoot weight, dry shoot weight and K^+^/Na^+^ ratio than H30 under salt stress. Under salt stress, the fresh shoot weight of DH20 was 60.21% and 70.92% higher than H30 in M1 and M2 generations, respectively (Figure 2B,C). For dry shoot weight, we observed that DH20 was 44.42% and 52.12% higher than H30 under salt treatment in M1 and M2 generations, respectively (Figure 2D,E). The K^+^/Na^+^ content of DH20 leaves was 85.88% and 90.89% higher than that of the leaves of H30 under salt condition in M1 and M2 generations, respectively (Figure 2F,G). These results indicate that DH20 could keep a higher shoot weight and K^+^/Na^+^ ratio in response to salt treatment than H30.

### 2.3. Comparison of Photosynthetic Characteristics between H30 and DH20 under Control and Salt Conditions 

**Figure 3:**
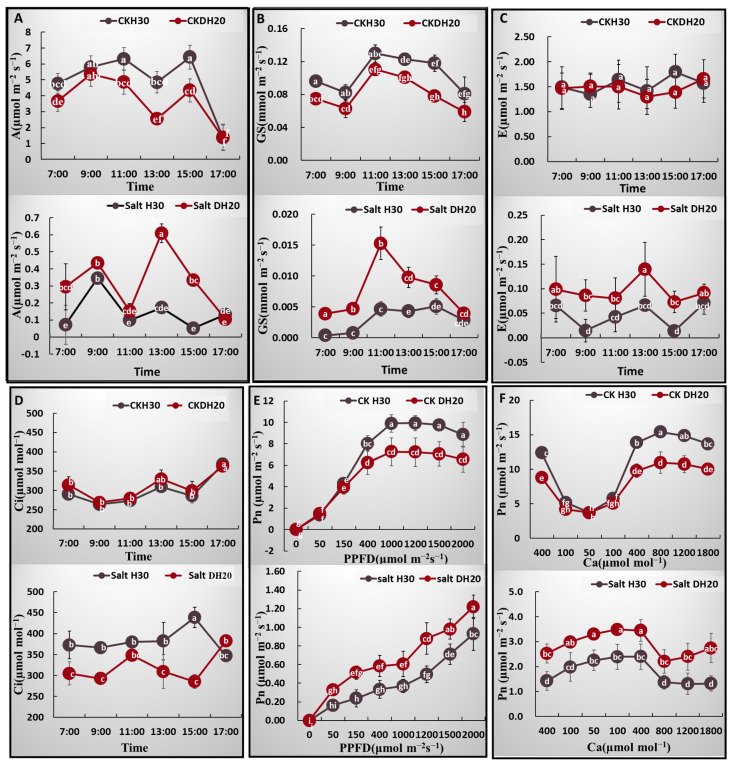
Comparison of photosynthetic characteristics between H30 and DH20 under CK and salt conditions. (**A**) Net photosynthetic rate (A), (**B**) stomatal conductance (Gs), (**C**) transpiration rate (E), (**D**) intercellular CO_2_ concentration (Ci), (**E**) response to photosynthetic photon flux density (PPFD), (**F**) response to ambient CO_2_ concentration (Ca). Different letters represent significant differences at 0.05 levels between DH20 and H30 under varying supply of time or PPFD or Ca (*n* = 10).

The photosynthetic characteristics, such as net photosynthesis (A), stomatal conductance (GS), transpiration (E) and intercellular CO_2_ concentration (Ci) were assessed to compare the salt tolerance between DH20 and H30. For the net photosynthesis rate (A), under the CK treatment, the A of H30 exceeded that of DH20 from 10:00 to 16:00. However, under the salt condition, the A of DH20 was significantly higher than that of H30 from 12:00 to 16:00 (Figure 3A). For the stomatal conductance (GS), the values for H30 were all significantly higher than those of DH20 from 07:00 to 17:00 under the CK condition, while the GS values of DH20 were higher than those of H30 from 07:00 to 16:00 under the salt treatment (Figure 3B). For transpiration (E), there were no significant differences between H30 and DH20 under the CK condition, while under salt treatment, the E value of DH20 exceeded that of H30 at the 09:00, 13:00 and 15:00 timepoints (Figure 3C). For intercellular CO_2_ concentration (Ci), similar to the transpiration (E), there were also no significant differences between H30 and DH20 under CK condition, but the value of Ci in H30 obviously exceeded that of DH20 under salt treatment at most timepoints, including 11:00, although this difference was not statistically significant at 11:00 (Figure 3D).

The light- and CO_2_-response curves of DH20 and H30 were quite similar. Under CK treatment, H30 showed a significantly higher Pn than DH20 when PPFD attained 400 μmol m^−2^ s^−1^ or more (Figure 3E,F). However, under the salt treatment, the Pn of DH20 was significantly higher than that of H30 when PPFD attained 150 μmol m^−2^ s^−1^ or more, while Ca was higher in DH20 at all time points (Figure 3E,F).

Based on the fitted light- and CO_2_-response curves, the light saturation point (LSP), apparent quantum efficiency (AQE), maximum net photosynthetic rate (Amax), maximum rate of RuBP carboxylation (V_cmax_) and maximum rates of electron transport (J_max_) were calculated and are presented in Supplementary Table S1. DH20 showed higher values of LSP and Amax than H30 under both CK and salt conditions. There was no significant difference in AQE between H30 and DH20, whether under CK or salt treatment. The performance of V_cmax_ and J_max_ in H30 were both significantly higher than DH20 under CK condition, while when treated with salt, DH20 showed significantly higher value than H30. The Fv′/Fm′ (the maximal photochemical efficiency of PSII in light) in DH20 was 27.11% greater than that of H30 under salt condition, while there was no significant difference in Fv′/Fm′ between DH20 and H30 under CK condition. 

### 2.4. Differentially Expressed Genes of DH20 in Response to Salt Stress 

To investigate the molecular basis of DH20 response to salt stress, we performed transcriptomic analysis of the leaves of H30 and DH20 using RNA-Seq on the Illumina HiSeq X Ten platform. A total of 46.54–85.30 M raw reads were obtained from the sequencing. After trimming the low-quality reads and adapters, we obtained 58.53 M, 50.96 M, 57.78 M and 59.49 M clean reads from samples of CK DH20, CK H30, salt DH20 and salt H30, respectively. The results of data quality assessment showed that clean reads exhibited good quality scores with a Q30 higher than 84.22% and the GC contents ranged from 54.94–57.41%. These results indicated that the quality was sufficient for further analysis. Moreover, the total reads ranging from 95.02% to 95.96% were mapped to the reference genome (Supplementary Table S2).

**Figure 4:**
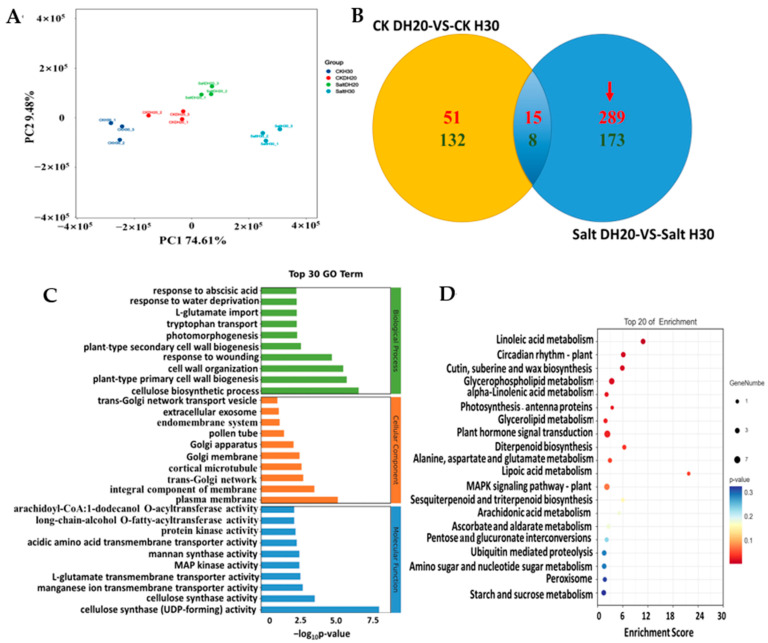
Analysis of specific differentially expressed genes (DEGs). Principal component analysis (PCA) (**A**) and Venn diagrams (**B**) of DEGs in H30 and DH20 under control and salt stress conditions. Up- and down-regulated DEGs are shown in red font and green font, respectively. Red arrow represents the specified DEGs in DH20. (**C**) Gene Ontology (GO) analysis. The gene ontology analysis of biological processes (green), cellular component (orange) and molecular function (blue) are indicated on the *y*-axis; −log_10_*p*-value is indicated on the *x*-axis. (**D**) Kyoto Encyclopedia of Genes and Genomes (KEGG) analysis. The *p* value is presented in a color scale; the size of the dots represents DEG number mapped in each pathway.

DEGs were identified by comparing the data for DH20 and H30 under salt and CK treatments. Principal component analysis (PCA) showed that the three replicates of each sample clustered together, indicating the good quality and repeatability of each biological replicate. There is a clear separation between CK and salt conditions in the first principal component, which accounts for 74.61% of the total variance (Figure 4A). Venn diagram analysis revealed that a total of 462 (289 up- and 173 down-regulated) genes were differentially regulated specifically in salt DH20 vs. salt H30; 23 DEGs (15 up- and 8 down-regulated) were the same between DH20 and H30; and 183 DEGs (51 up- and 132 down-regulated) were differentially detected specifically in CK DH20 vs. CK H30 (Figure 4B). It is indicated that DH20 performed better by up- or down-regulating more DEGs throughout the stress conditions. This result also supports the finding that DH20 was more tolerant to salt than H30 at the transcriptome level. To get a better view of the transcriptomic profiles in response to the salt stress, a gene expression cluster analysis-based heatmap of 462 DEGs was produced and is presented in Supplementary Figure S2. 

To better understand the functions of the 462 DEGs, they were functionally categorized using GO and KEGG enrichment analyses. GO functional analysis showed that 91 GO terms were significantly enriched (FDR (false discovery rate) ≤ 5; no. of DEGs in one GO term ≥ 3), including 51 biological process, eight cellular components and 32 molecular functions. For the biological process, the regulated DEGs were significantly enriched in “cellulose biosynthetic process (GO:0030244)” (*p* = 1.03 × 10^−7^). For the cellular component, the DEGs were significantly enriched in “plasma membrane (GO:0005886)” (*p* = 3.18 × 10^−6^). For the molecular function, DEGs were significantly enriched in “cellulose synthase (UDP-forming) activity (GO:0016760)” (*p* = 3.56 × 10^−9^) (Figure 4C). KEGG analysis revealed that there were 462 DEGs mapped to 45 KEGG pathways, in which 11 KEGG pathways were significantly enriched. The 11 enriched pathways were linoleic acid metabolism (ko00591); circadian rhythm-plant (ko04712); cutin, suberine and wax biosynthesis (ko00073); glycerophospholipid metabolism (ko00564); alpha-linolenic acid metabolism (ko00592); photosynthesis-antenna proteins (ko00196); glycerolipid metabolism (ko00561); plant hormone signal transduction (ko04075); Diterpenoid biosynthesis (ko00904); alanine, aspartate and glutamate metabolism (ko00250); and lipoic acid metabolism (ko00785) (Figure 4D).

### 2.5. Differentially Abundant Metabolites of DH20 in Response to Salt Stress

**Figure 5:**
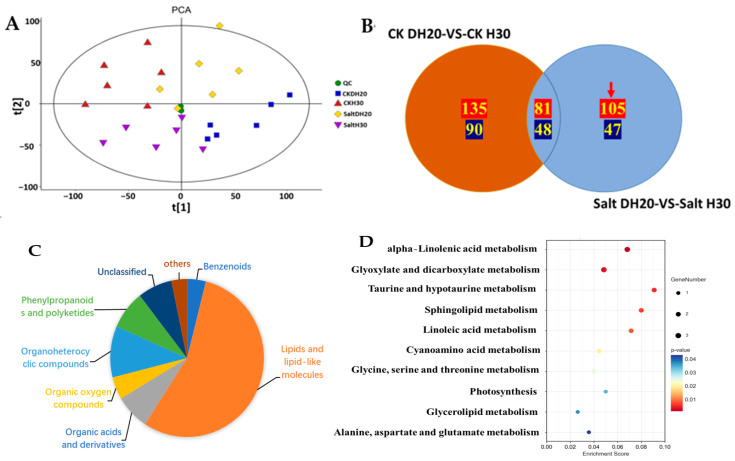
Analysis of DAMs in H30 and DH20 under control and salt-stress conditions. (**A**) Principal component analysis (PCA) of metabolites in DH20 and H30 under CK and salt conditions. (**B**) Venn diagram. Positive and negative DAMs are shown in red font and green font, respectively. Red arrow represents the specified DAMs in DH20. (**C**) Proportion of different super classes in 152 metabolites. The different colors in each pie chart indicate different classifications, and the different areas represent the relative proportion of metabolites in the classification; (**D**) Kyoto Encyclopedia of Genes and Genomes (KEGG) analysis of 152 differentially accumulated metabolites.

To construct a systematic profile of metabolic changes that occur in response to salt stress, untargeted metabolomic analysis was performed to compare and contrast H30 and DH20, using the same samples used for the transcriptomic analysis. Principal component analysis (PCA) was performed (Figure 5A,B). PCA showed that R2X [1] = 0.205 and R2X [2] = 0.126 of the total variance, and that QC samples were closely clustered together. It is indicated that the biological replicates grouped together, and that the metabolomic data had good stability and repeatability.

A total of 3709 metabolites were identified, of which 2474 were positive and 1235 were negative (Supplementary Figure S3). DAMs (Differential Accumulation Metabolites) were screened with a standard of *p* < 0.05 and VIP > 1. In total, 225 DAMs (135 positive and 90 negative in ion mode) were identified from the comparison of CK DH20 vs. CK H30, of which 129 DAMs (81 positive and 48 negative) were the same as the comparison of salt DH20 vs. salt H30, and 152 DAMs (105 positive and 47 negative) were specifically detected in DH20 and used for further analysis (Figure 5B). These 152 metabolites belonged to eight super classes and 40 classes, with the top belonging to fatty acyls (42 in total, including 15 fatty acids and conjugates, eight eicosanoids, four fatty acyl glycosides, four fatty amides, four fatty esters, three octadecanoids, two fatty alcohols, one docosanoids and one other fatty acyls), steroids and steroid derivatives (11 in total, including four steroidal glycosides; three bile acids, alcohols and derivatives; one cycloartanols and derivatives; one estrane steroids; one steroidal alkaloids and one androstane steroids), prenol lipids (nine in total, including four triterpenoids, one diterpenoids, one monoterpenoids, one sesquiterpenoids, one sesquiterpenoids and one terpene lactones), carboxylic acids and derivatives (eight in total, including one carboxylic acid derivatives and seven amino acids, peptides and analogues), organooxygen compounds (seven in total, including six carbohydrates and carbohydrate conjugates and one alcohol or polyol), and glycerophospholipids (seven in total, one CDP-glycerols, two glycerophosphocholines, one glycerophosphoethanolamines, one glycerophosphoglycerols and two glycerophosphoserines) (Figure 5C and Supplementary Figure S4). 

The KEGG analysis of these specific DAMs in DH20 showed that these DAMs were significantly involved in 10 pathways (*p* < 0.05). The top significant enriched pathways were alpha-linolenic acid metabolism (ats00592), glyoxylate and dicarboxylate metabolism (ats00630), taurine and hypotaurine metabolism (ats00430), sphingolipid metabolism (ats00600), linoleic acid metabolism (ats00591), cyanoamino acid metabolism (ats00460) and glycine, serine and threonine metabolism (ats00260) (Figure 5D). 

### 2.6. Association Analysis of DEGs and DAMs in Response to Salt Stress

**Figure 6:**
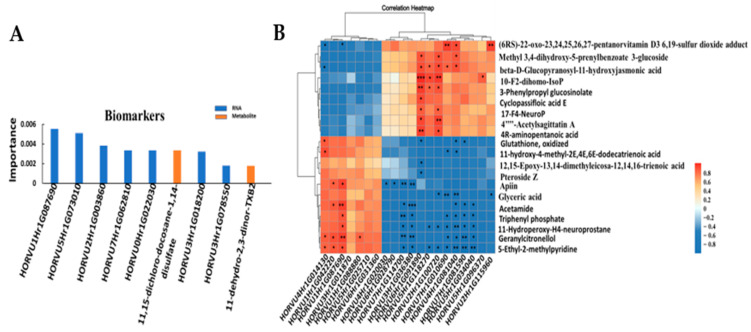

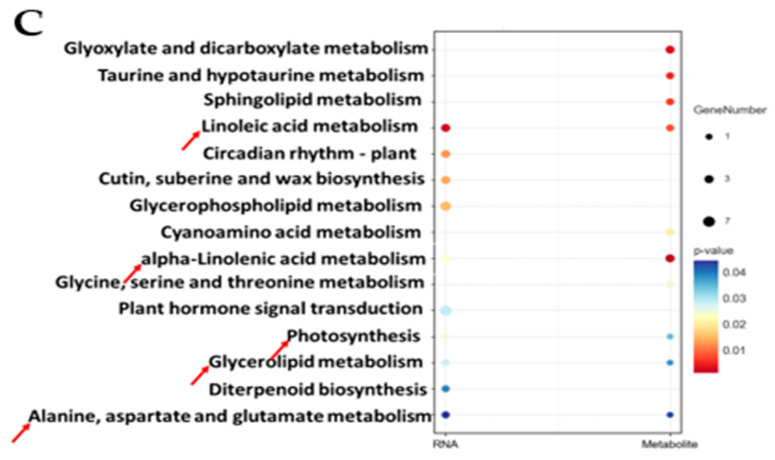
Association analysis of genes and metabolites. (**A**) key biomarkers; (**B**) correlation. The top 20 differential genes are on the *x*-axis, and the top 20 differential metabolites are on the *y*-axis. * means correlation, *p* < 0.05; ** means correlation, *p* < 0.01; and *** means correlation, *p* < 0.001. (**C**) Integrated analysis of KEGG enrichment of TOP20 of DEGs and DAMs. The horizontal coordinate is different omics, the vertical coordinate is the pathway, the bubble size represents the number of different pathways, and the bubble color represents the *p*-value of the pathway, while the red arrows indicate the further analysis pathways.

According to the combined transcriptome and metabolites analysis, nine key biomarkers, including two metabolites and seven genes, were identified; that is, 11,15-dichloro-docosane-1,14-disulfate,11-dehydro-2,3-dinor-TXB2, *HORVU1Hr1G087690* (ubiquitin 5), *HORVU5Hr1G073010* (nucleolar protein gar2-related isoform 5), *HORVU2Hr1G003860* (receptor kinase 1), *HORVU7Hr1G062810* (GTPase Era), *HORVU0Hr1G022030* (VQ motif family protein), *HORVU3Hr1G018200* (pentatricopeptide repeat (*PPR*) superfamily protein) and *HORVU3Hr1G078550* (basic-leucine zipper (*bZIP*) transcription factor family protein) (Figure 6A). 

The significantly differential metabolites and genes of the top 20 (*p* value < 0.05) were selected to investigate the correlation between the 152 specific DAMs and 462 specific DEGs. The correlated heatmap showed that the top three correlated DAMs were 5-ethyl-2-methylpyridine, geranylcitronellol and 11-hydroperoxy-H4-neuroprostane, which were correlated with 11 DEGs, 10 DEGs and 9 DEGs, respectively (Figure 6B). The top three correlated DEGs were *HORVU4Hr1G081040* (ABC transporter G family member 45), *HORVU6Hr1G091890* (aconitate hydratase 1) and *HORVU2Hr1G100720* (unknown), which were correlated with 11 DAMs, 11 DAMs and 8 DAMs, respectively.

KEGG analysis revealed that the five most significant common enrichment pathways were linoleic acid metabolism, alpha-linolenic acid metabolism, glycerolipid metabolism, photosynthesis, and alanine, aspartate and glutamate metabolism (Figure 6C). Based on this, these metabolic pathways were selected for further analysis.

### 2.7. Improving Phosphatidylcholine in the Lipid Metabolism of DH20 in Response to Salt Stress

**Figure 7:**
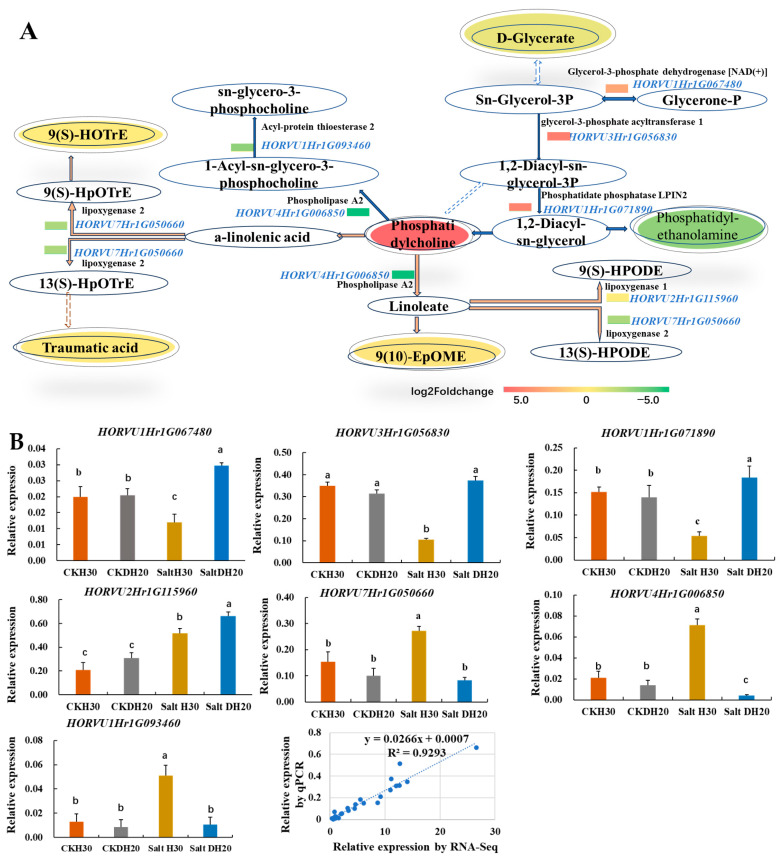
Overview of part of linoleic acid, alpha-linolenic acid and glycerolipid metabolism in response to salt stress. (**A**) Pathway. The oval represents the metabolites and the significantly up- and down-regulated metabolites at *p* < 0.05; those that are VIP > 1 are indicated in colored fonts. The changed DEGs are highlighted by blue font, and the colored rectangle before DEGs represents the value of the log2 foldchange (salt DH20 vs. salt H30). The solid and dotted lines with arrows indicate direct or indirect interactions of the processes, respectively. The orange lines indicate processes in linoleic acid and alpha-linolenic acid metabolism, and the blue lines indicate glycerolipid metabolism. (**B**). Validation of seven DEGs by qPCR. The X axes represent the barley samples under CK conditions and salt treatment, and the Y axes represent the normalized relative quantity of gene expression. The normalized relative quantity (NRQ) is calculated as a target gene’s expression with respect to two endogenous reference genes: *HvGAPDH* and *HvActin*. Different letters indicate significant differences at the 0.05 level by Student’s *t* test. All data represent three biological replicates. Correlation analysis of the seven DEGs by qPCR and RNA-seq is also shown, and the Pearson correlation coefficient (R^2^) is 0.9293.

In DH20 leaves under salt stress, six DAMs and seven DEGs were enriched, and these were involved in linoleic acid metabolism, alpha-linolenic acid metabolism and glycerolipid metabolism. The up-regulated expression of *HORVU1Hr1G067480* (glycerol-3-phosphate dehydrogenase [NAD(+)]), *HORVU3Hr1G056830* (glycerol-3-phosphate acyltransferase 1) and *HORVU1Hr1G071890* (phosphatidate phosphatase LPIN2) should enhance the conversion of glycerone-P into phosphatidylcholine, whose content was significantly elevated. The reduction in 9(S)-HOTrE, traumatic acid, 9(10)-EpOME and D-glycerate might be due to the down-regulated expression of *HORVU7Hr1G050660* (lipoxygenase 2) and *HORVU4Hr1G006850* (phospholipase A2). Besides, we also observed the down-regulated expression of *HORVU1Hr1G093460* (acyl-protein thioesterase 2), *HORVU2Hr1G115960* (lipoxygenase 1) and *HORVU7Hr1G050660* (lipoxygenase 2), which may lead to the reduction in sn-glycero-3-phosphocholine, 9(S)-HPODE and 13(S)-HPODE (Figure 7A). 

We confirmed the expression of seven DEGs by q-PCR. The q-PCR determination shows that the expression of *HORVU1Hr1G067480*, *HORVU3Hr1G056830*, *HORVU1Hr1G071890* as well as *HORVU2Hr1G115960* was up-regulated in *DH20* when comparing to H30 under salt condition, while *HORVU7Hr1G050660*, *HORVU4Hr1G006850* and *HORVU1Hr1G093460* were down-regulated. The correlation coefficient between qPCR and RNA-seq results was 0.9293, confirming the validation of RNA-seq data (Figure 7B).

### 2.8. Maintaining ADP and Decreasing L-glutamate of DH20 in Response to Salt Stress 

**Figure 8:**
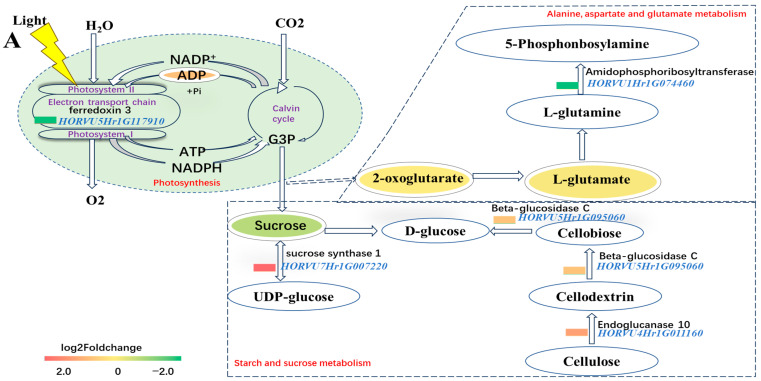

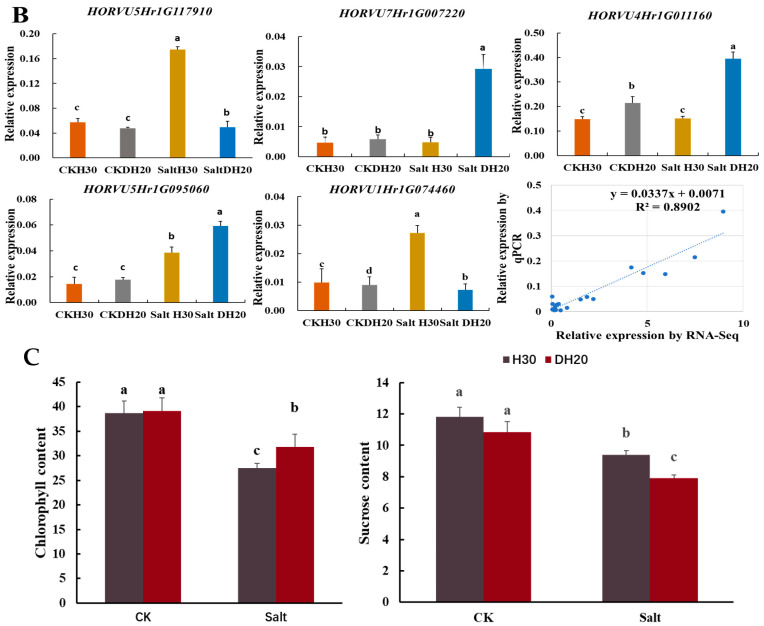
Overview of part of photosynthesis and alanine, aspartate and glutamate metabolism in response to salt stress. (**A**). Pathway. The ovals represent the metabolites and the significantly up- and down-regulated metabolites at *p* < 0.05, and those that are VIP > 1 are indicated in colored fonts. The changed DEGs are highlighted by blue font, and the colored rectangle before DEGs represents the value of the log2 foldchange (salt DH20 vs. salt H30). The solid and dotted lines with arrows indicate direct or indirect interactions of the processes, respectively. The dotted boxes indicate the alanine, aspartate and glutamate metabolism as well as starch and sucrose metabolism. The green oval indicates photosynthesis. (**B**) Validation of five DEGs by qPCR. The X axes represent the barley samples under CK conditions and salt treatment, and the Y axes represent the normalized relative quantity of gene expression. The normalized relative quantity (NRQ) is calculated as a target gene’s expression with respect to two endogenous reference genes: *HvGAPDH* and *HvActin*. Different letters indicate significant differences at the 0.05 level by Student’s *t* test. All data represent three biological replicates. Correlation analysis of the five DEGs by qPCR and RNA-seq is also shown, and the Pearson correlation coefficient (R^2^) is 0.8902. (**C**) Chlorophyll content and sucrose content validation. Different lowercase letters indicate significant differences at the 0.05 level between H30 and DH20 by Student’s *t* test. The error bars are shown from the analysis of five biological replicates.

In light reactions of photosynthesis, ADP(C00008) was the metabolite that significantly increased in DH20 when treated with the salt stress, while the gene *HORVU5Hr1G117910* (ferredoxin 3) was down-regulated. Glyceraldehyde 3-phosphate (G3P) is a crucial component of the Calvin cycle for converting light energy into carbon compounds. The reduction in sucrose in DH20 under salt condition might be due to the conversion into UDP-glucose. The upregulated expression of *HORVU4Hr1G011160* (endoglucanase 10) and *HORVU5Hr1G095060* (beta-glucosidase C) might accelerate D-glucose accumulation. In alanine, aspartate and glutamate metabolism, although the content of 5-phosphonbosylamine did not change substantially, two DAMs (2-oxoglutarate and L-glutamate) and gene *HORVU1Hr1G074460* (amidophosphoribosyltransferase) were found to be reduced (Figure 8A). 

To confirm the validity of the RNA-seq-based transcript abundance of genes, five DEGs involved in the pathway were analyzed (Figure 8B). The data confirmed that the up-regulation of three genes (*HORVU7Hr1G007220*, *HORVU4Hr1G011160* and *HORVU5Hr1G095060*) were found in DH20 when compared to H30 under salt stress. Two genes (*HORVU5Hr1G117910* and *HORVU1Hr1G074460*) were down-regulated in DH20 compared to H30 under salt stress. The results showed that the expression patterns of these genes were consistent with the RNA-seq data (R^2^ = 0.8902) (Figure 8B). 

To confirm the photosynthetic metabolism difference between H30 and DH20 under the salt stress condition, the content of chlorophyll and sucrose were also analyzed. Chlorophyll content in DH20 showed a significant increase (15.49%) under salt treatment compared to H30 but no significant difference under the CK condition (Figure 8C). For sucrose content, the results showed that the content of DH20 was significantly (15.85%) lower than that of H30 under salt condition (Figure 8C). 

## 3. Discussion

### 3.1. Rapid Production of DH Lines for Salt Tolerance by Mutagenesis Combined with Microspore Culture Technology

Mutagenesis combined with microspore culture technology has many advantages in plant breeding. Firstly, compared to anther culture, isolated microspore culture is an ideal way to produce DH lines, without interference from heterozygous diploid somatic cells from anther wall tissues [11]. Secondly, mutagenesis combined with microspore culture can be used to improve breeding efficiencies by stabilizing genetic variations quickly, especially in the selection of traits controlled by recessive genes [18,19]. Furthermore, it is a feasible and cost-effective approach in early selection to use mutagenetic materials under controlled laboratory conditions in comparison with field selection [20]. 

In this study, a homozygous line (DH20) with improved salt tolerance compared with its wild type was rapidly generated by mutagenesis combined with microspore culture. The shoot fresh weight and shoot dry weight in M1 and M2 generations showed that the performance of DH20 was heritable and stably superior to the wild type H30 under high concentrations of NaCl treatment (360 mmol). In general, barley, as one of the most salt-tolerant cereal crops, can grow in up to 250–300 mM NaCl [21,22]. Compared to H30, a higher K^+^/Na^+^ ratio in shoots of DH20 might be one of the factors attributing to the difference in shoot growth under the severe salt condition. It has been reported that shoot growth is more sensitive to salt stress than root [23]. Previous studies have also shown that sodium/potassium ion homeostasis plays an important role under high salinity [24].

Consequently, mutagenesis combined with microspore culture could be applied to rapidly fix superior traits, including salt tolerance, and produce new germplasm in a completely homozygous state, as has been used in nitrogen-use-efficiency improvement [12,25]. This study provides an alternative way to produce mutant lines for salt tolerance in future crop breeding. Gene mining to elucidate the molecular mechanisms that determine how DH20 copes with salinity is worthy of further study.

### 3.2. Lipid Metabolism Underlying the DH20 Response to Salt Stress

Recent studies have also drawn attention to the importance of lipid signaling in plant responses to abiotic stress [26]. The main classes of lipids include fatty acyls, glycerolipids and glycerophospholipids [27]. Fatty acyls participate not only in the regulation of energy storage substances and cell membrane plasm composition, but also in plant resistance to adversity [27]. In this study, fatty acids (15) were the most prominent type of fatty acyls (42) accumulated in DH20 under salt stress. The combined transcriptome and metabolome analysis showed that 11,15-dichloro-docosane-1,14-disulfate (grouped within ‘other fatty acyls’) and 11-dehydro-2,3-dinor-TXB2 (eicosanoids) were two key biomarkers that distinguished DH20 and H30 under the salt treatment. Some studies have shown that the enhancement of stress tolerance is due to changes in membrane fluidity caused by fatty acid desaturation, which can prevent membrane hardening/damage caused by stress and maintain the structural and functional integrity of cell membranes [28,29]. It has also been demonstrated that unsaturated fatty acids can serve as a sink for ROS and can immediately scavenge ROS through non-enzymatic reactions without activating gene expression or signaling pathways [28,29]. However, as yet, there are few studies about the effect of eicosanoids on salt resistance of plants, especially in barley.

Further, the combined pathway analysis in this study suggested that linoleic acid metabolism, alpha-linolenic acid metabolism and glycerolipid metabolism play important roles in DH20 to cope with salt stress. It has been reported that linoleic acid metabolism may be important for melatonin-mediated salt tolerance in banana [30] and rice [31]. Furthermore, compound microbial inoculants have been shown to promote the growth of wheat under salt stress by regulating the expression of linoleic acid metabolism [32]. Another study showed that linolenic acid in the plasma membranes of barley roots increased under salt stress [33]. However, to our knowledge, this is the first report on these three pathways of salt tolerance in barley leaves, especially in mutants derived from the mutagenesis combined with microspore culture. 

In the lipid metabolism pathway of this study (Figure 7), when DH20 was exposed to the salt stress, three DEGs (*glycerol-3-phosphate dehydrogenase [NAD*(*+*)*]*, *glycerol-3-phosphate acyltransferase 1* and *phosphatidate phosphatase LPIN2*) were upregulated, while four DEGs (*lipoxygenase 1*, *lipoxygenase 2, phospholipase A2* and *acyl-protein thioesterase 2*) were down-regulated. There was a decrease in the content of 9(S)-HOTrE, traumatic acid, D-glycerate and 9(10)-EpOME. 9(10)-EpOME may eventually lead to the accumulation of the phosphatidylcholine (PC), and it has been reported that phosphatidylcholine shows an overall reduction in salt-treated barley roots [34]. Some studies have also revealed that phosphatidylcholine enhances homeostasis in peach seedling cell membrane or in *Arabidopsis thaliana* in response to salt stress [35,36]. The increase in phosphatidylcholine in DH20 leaves under salt stress might have effects on lipid homeostasis, which could sustain cell expansion and the growth of salt-stressed plants [37].

Taken together, the accumulation of lipids in DH20 when exposed to salt, especially unsaturated lipids, may not only alleviate membrane damage caused by salt stress but may also scavenge ROS and prevent the oxidization of other cell components in salt-tolerant varieties. Besides, it is speculated that the lipid layers in the intracellular membranes of DH20 might play key roles in regulating the flux of K^+^ and Na^+^ ions across membranes. As previously reported, different lipid compositions may correlate with different activities in cross-membrane ion transport [38,39]. In future, the distinct membrane lipid compositional differences between DH20 and H30 in salinity response could be further elucidated.

### 3.3. Adaptive Strategies of Photosynthesis Underlying the DH20 Response to Salt Stress

Photosynthesis is often the first process affected by abiotic stresses [40]. Here, diurnal variation in photosynthetic characteristics (A, GS, E and Ci) of DH20 were better than those of H30 under salt-stress conditions through most of the diurnal cycle, which might be due to stomatal factors. A similar result was also found by Shi etal [41] in *Hordeum jubatum*. Other important photosynthetic parameters (V_cmax_ and J_max_) from the light- and CO_2_-response Curves were also significantly higher in DH20 than H30 when treated to salt, indicating that DH20 was more active in carbon assimilation under salt stress. Moreover, chlorophyll has a crucial role in photosynthesis, light harvesting and light energy transduction. Previous studies have found that plants with a lower chlorophyll content are more susceptible to photoinhibition under abiotic stresses [42]. Higher chlorophyll content in DH20 means less photosynthesis inhibition after salt stress (Figure 8C). 

We also found that photosynthesis was the significant enriched pathway through KEGG analyses of the transcriptome and metabolism results. Here, a significant increase in the ADP of DH20 was observed under salt condition, while pet F (*HORVU5Hr1G117910*, ferredoxin3) showed a decrease. This phenomenon may lead to the decline of ATP/ADP. It is reported that comparatively lower ATP/ADP ratios in *S. portulacastrum* compared to *B. juncea* suggest the better utilization of energy produced to support different processes to cope with salt tolerance [43]. Wu et al. [44] also observed that increasing glycolysis and energy consumption during the most stressful time under high-salinity stress is a tolerance mechanism in cultivated barley. As previously reported, sucrose is the primary soluble sugar that can be altered by salinity, and it also acts as a key osmo-protectant and signaling molecule in response to stress [45,46]. Under severe salt stress, sugars may decrease and could modify gene expression patterns, namely those main governing photosynthetic metabolism [40]. The reduced levels of sucrose are crucial for plants when adapting to salt stress [47]. A similar result was also found in this study: two genes, *HORVU4Hr1G011160* (endoglucanase 10) and *HORVU5Hr1G095060* (beta-glucosidase C), were up-regulated, and sucrose content declined. This result was also confirmed by the determination of sucrose content. 

It is speculated that the efficient management of energy consumption might be a key strategy for DH20 to tolerate salt stress. For example, DH20 might tend to save energy through limiting amino acid metabolism. Two metabolites (2-oxoglutarate and L-glutamate) and one gene (*HORVU1Hr1G074460*; amidophosphoribosyltransferase) involved in alanine, aspartate and glutamate metabolism related to the antioxidant system were significantly down-regulated, indicating that reducing the accumulation of ROS is one of the adaptive mechanisms for DH20 resistance to high salt stress. A study also demonstrated that concentrations of free amino acids might contribute to plant adaptive responses to salinity by regulating K^+^ transport across the plasma membrane, thus enabling the maintenance of an optimal K^+^/Na^+^ ratio [48]. 

### 3.4. Molecular Biomarkers Underlying the DH20 Response to Salt Stress 

In this study, seven DEGs were identified as biomarkers to distinguish DH20 and H30 under the salt stress condition. One gene (*HORVU1Hr1G087690*) encoding ubiquitin 5, was significantly down-regulated in DH20 compared to H30 when treated with salt stress. As previously reported, the ubiquitin-proteasome system has a critical role in regulating plant abiotic stress responses [49,50]. A report has shown that the ubiquitin-binding protein OsDSK2a could coordinate seedling growth and salt responses by regulating gibberellin metabolism in rice [51].

A VQ motif family protein has been shown to participate in plant abiotic stress responses in many species, including tea [52] and Arabidopsis [53]. It can interact with WRKY transcription factors via conserved V and Q residues to regulate plant growth. Here, one gene encoding a VQ motif family protein (*HORVU0Hr1G022030*) showed down-regulation in DH20 under salt condition.

The pentatricopeptide repeat (*PPR*) superfamily is one of the largest gene families in plants, which mainly regulates organelle RNA metabolism. Accumulating evidence has demonstrated that *PPR* plays a crucial role in plants to respond to biotic or abiotic stress [54]. For example, *AtPPR96* is involved in the response of plants to salt stress [55]. In this study, the gene *HORVU3Hr1G018200* encoding a PPR superfamily protein was up-regulated in DH20 when exposed to salt.

Basic leucine zipper (bZIP) transcription factors are the most conserved and widely distributed transcription factors in plants. bZIPs have been found to be involved in salt-stress tolerance in *Oryza sativa* [56], Arabidopsis [57] and *Lycopersicon esculentum* [58]. Several studies on the mechanism of action of bZIP transcription factors have found that they can interact with ABRE, PB, GLM, G-box, H-box and ACEs (ACGT) elements to induce the expression of downstream genes. The expression of these downstream genes can increase the activity of SOD, CAT, and POD; reduce the content of H_2_O_2_ and MDA; and improve the ability of plants to tolerate salt stress [59]. We found a gene (*HORVU3Hr1G078550*)-encoding bZIP transcription factor family protein to be down-regulated in DH20 under salt conditions. 

Receptor-like protein kinases, a large group of protein kinases in plants, play key roles in stress-sensing and signal transduction under different stress conditions [60]. The function of RPK1 has been characterized in Arabidopsis and shown to be expressed in the flowers, stem, leaves and roots, and to be rapidly induced by salt and other stresses. It is suggested that the *RPK1* gene is involved in general abiotic stress responses [61]. A similar result was also found in this study in that *HORVU2Hr1G003860* (receptor kinase 1) showed upregulation in DH20 under salt conditions. 

In plants, GTPase Era was first found in *Antirrhinum majus* and is identified in dividing or metabolically active cells. GTPase Era was predicted to target to mitochondria through N-terminal amino acid sequences, and a deletion allele of GTPase Era created by site-selected transposon mutagenesis leads to the stunted development of seeds containing embryos and endosperm after fertilization [62]. Prior studies also showed that ERA is important for pleiotropic processes, including adaptation to thermal stress, fatty acid metabolism and carbon metabolism [63]. We found that the gene *HORVU7Hr1G062810,* encoding GTPase Era, was down-regulated, while another gene (*HORVU5Hr1G073010*) encoding nucleolar protein gar2-related isoform 5 was up-regulated in DH20 compared to H30 under salt conditions. To date, there are few reports on the role of GTPase Era and nucleolar protein gar2-related isoform 5 in the response to salt stress. Taken together, the results suggest that these seven genes are pivotal players for DH20 to cope with salt stress. In future, it would be interesting and meaningful to identify the causative mutations responsible for better salt tolerance in DH20 by DNA sequencing. 

## 4. Materials and Methods

### 4.1. Plant Materials and Isolated Microspore Culture

Barley (*Hordeum vulgare* L.) variety H30 was used as the wild type (WT). This variety is the one primarily cultivated in the Yangtze River Delta of China. The mutant materials were obtained by treating dry seeds of H30 with 500 Gy Co-60 gamma irradiation, followed by microspore culture. WT and mutated seeds were obtained from the Biotechnology Research Institute, Shanghai Academy of Agriculture Sciences, China.

Microspores were isolated following the procedures described by [64]. Briefly, the middle florets of an inflorescence of the spikes with early to mid-uninucleate microspores were collected and subjected to cold pretreatment at 4 °C for 15 days. After the cold pretreatment, the spikes were sterilized with 75% ethanol, and then the sterilized spikes were blended in an ultra-speed blender in an extraction buffer comprising 5 mM MES hydrate, 10 mM calcium chloride (CaCl_2_) and 330 mM mannitol (Sigma–Aldrich, Burlington, MA, USA). Microspores were collected and the density adjusted to 1.1 × 10^5^ mL^−1^ before being placed in the dark at 25 °C for 2 days for embryogenic calli induction. The induction medium contained N6 basal medium supplemented with 0.25 M maltose, 2.3 µM kinetin (KT) and 2.0 µM 2.4-D. After 21 d of culture, the induced embryogenic calli were transferred to a differentiation medium for plant regeneration. This comprised 9.5 mM agar-solidified MS supplemented with 7.0 µM KT, 0.3 µM NAA, 88 mM maltose and 2.2 µM 6-BA. The growth conditions for the regenerated green seedlings were 25 °C with a 16 h photoperiod under light of 150 µmol m^−2^ s^−1^. Finally, the ploidy of the regenerated plantlets was determined by flow cytometry, and then the regenerated doubled haploid (DH) plantlets were transferred to Kunming (Yunnan province, China) to harvest the seeds for producing the DH lines [25].

### 4.2. Hydroponic Culture Screening

Seeds of H30 and mutated plants were firstly surface-sterilized with 10% NaClO for 10 min. Then, the seeds were germinated on wet filter paper in a Petri dish for 7 days. After that, the seedlings were grown hydroponically using Hoagland solution in a controlled environment with a 16/8 h light/dark cycle at 22 °C/18 °C (light/dark). The light intensity was 300 μmol m^−2^ s^−1^ with a 16 h photoperiod and relative humidity approximately 70%, according to Xu et al. [65]. Until the two-leaf stage, plants were randomly divided into two groups, one denoted as control (CK), the other salt treated with 360 mmol/L NaCl as the salt treatment. Plants were supplied with fresh nutrient solution every three days, and the pH was adjusted to 6.2 ± 0.5. 

### 4.3. Determination of Shoot Fresh Weight, Shoot Dry Weight, Shoot K^+^/Na^+^ Content and Leaf Photosynthetic Parameters 

After 28 d of salt treatment, seedlings were sampled for the determination of fresh weight, dry weight and the K^+^/Na^+^ ratio. Fresh weight of shoots was weighed immediately after sampling. The samples were then treated in an oven at 105 °C for 30 min and dried at 70 °C to obtain the stable dry weight. The contents of Na^+^ and K^+^ were determined by an inductively-coupled argon plasma emission spectrometer iCAP6300 instrument (Thermo Scientific, Walthmam, MA, USA). Six biological replicates were used for each measurement.

After 2 days exposure to salt treatment, the second fully expanded leaves counting from the top of the stem, at the same position in both control and salt-treated plants, were used to determine photosynthetic parameters in each case with ten independent biological replicates. The chlorophyll content was measured using a chlorophyll meter (SPAD-502; Minolta, Tokyo, Japan). The net photosynthetic rate (A), stomatal conductance (gsw), intercellular CO_2_ concentration (Ci), transpiration rate (E) and Fv’/Fm’ were measured using a Li-6800 portable photosynthesis system (LI-CORInc., Lincoln, Dearborn, MI, USA). The diurnal variations of photosynthesis were determined under natural light intensity on a sunny day every two hours from 07:00 to 17:00. The light-response curves and CO_2_-response curves were constructed with data collected as described by Cao et al. [66]. The other key photosynthetic parameters were calculated from the curve, including maximum net photosynthetic rate (Pmax), light saturation point (LSP), light compensation point (LCP), apparent quantum yield (AQY), maximum rate of RuBP carboxylation (V_cmax_) and maximum rates of electron transport (J_max_).

### 4.4. RNA Extraction, Library Construction, Illumina Sequencing and Transcriptome Analysis

The seedlings were collected after 2 d of hydroponic cultures with CK and salt supply for RNA isolation. Three individuals were pooled as one biological replicate. All the RNA-seq was performed at the Shanghai OE Biotech. Co. Ltd., Shanghai, China. In brief, total RNA was extracted using the mirVana miRNA Isolation Kit (Ambion, Austin, TX, USA). The quality and integrity of the RNA were determined using a NanoDrop spectrophotometer (ThermoFisher Scientific, Waltham, MA, USA) and Agilent 2100 Bioanalyzer (Agilent, Santa Clara, CA, USA). The samples with an RNA integrity number (RIN) ≥ 7 were selected for the subsequent analysis. cDNA libraries were constructed using the TruSeq Stranded mRNA LT Sample Prep Kit (Illumina, San Diego, CA, USA), according to the instructions of the manufacturer. Then, four independent cDNA libraries were sequenced on the Illumina HiSeq X Ten sequencing platform (Shanghai OE Biotech. Co. Ltd., Shanghai, China), and 150 bp paired-end reads were generated.

Clean reads were obtained by removing adapters, low-quality reads with Q < 20, or ambiguous bases (‘N’). The resulting clean reads were mapped to the reference genome of barley (http://plants.ensembl.org/Hordeum_vulgare/Info/Index?db=core (accessed on 14 November 2019)). The methods for identification of DEGs and functional analysis were as described by Wang et al. [67]. Briefly, fragments per kb per million reads (FPKM) values for each gene were calculated using Cufflinks [68], and differential expression analysis was performed using the R package DESeq. DEGs with threshold *p*-values < 0.05 and |log2fold change| > 1) were identified for significantly differential expression [69]. A hierarchical cluster analysis of DEGs was performed to explore gene expression patterns. Venn diagram software (http://bioinfogp.cnb.csic.es/tools/venny/index.html (accessed on 20 November 2019)) was used to sort the DEGs. The enrichment analysis of the GO and KEGG pathways were performed using R (version 4.0.4) based on hypergeometric distribution [70]. 

### 4.5. Metabolite Profiling and Data Analysis

The plant materials used for metabolome analysis were the same as those used for transcriptome analysis. Six biological replicates were prepared for each sample, and in each case, 80 mg was weighed and extracted in 20 µL of 2-chloro-l-phenylalanine (0.3 mg/mL in methanol) and Lyso PC17:0 (0.01 mg/mL in methanol), and a 1 mL mixture of methanol and water (7/3, *v*/*v*). Samples were ground at 60 HZ for 2 min and ultrasonicated at ambient temperature for 30 min after vortexing, then placed at −20 °C for 20 min. Samples were then centrifuged at 13,000 rpm at 4 °C for 10 min. A mixture of methanol and water (400 μL, 1/4, *v*/*v*) was added to 300 µL of supernatant, and the samples vortexed for 30 s and then ultrasonicated for 2 min. Samples were then centrifuged at 13,000 rpm at 4 °C for 10 min. The supernatants (150 μL) from each tube were collected using crystal syringes, filtered through 0.22 μm microfilters and transferred to LC vials. The vials were stored at −80 °C for LC-MS analysis. Quality control (QC) samples were prepared by mixing the aliquots of all the samples into a pooled sample. 

The samples were placed in an ACQUITY UPLC system (Waters Corporation, Milford, MA, USA) coupled with an AB Triple TOF 5600 (AB SCIEX, Framingham, MA, USA) for metabolic profiling in both ESI-positive and ESI-negative ion modes. An ACQUITY UPLC BEH C18 column (1.7 μm, 2.1 × 100 mm) was employed in both positive and negative modes. The binary gradient elution system consisted of (A) water (containing 0.1% formic acid) and (B) acetonitrile (containing 0.1% formic acid), and separation was achieved using the following gradient: 0 min, 5% B; 2 min, 20% B; 4 min, 25% B; 9 min, 60% B; 17 min, 100% B; 19 min, 100% B; 19.1 min, 5% B; and 20.1 min, 5% B. The flow rate was 0.4 mL/min, and the injection volume was 5 μL. The parameters of mass spectrometry were as follows: curtain gas of 35 PSI; ion spray voltage, 5500 V (+) and 4500 V (−); ion source temperature, 550 °C (+) and 550 °C (−); declustering potential, 100 V (+) and −100 V (−); collision energy, 10 eV (+) and −10 eV (−); and interface heater temperature, 550 °C (+) and 600 °C (−). 

The raw data were collected using UNIFI 1.8.1 and analyzed using Progenesis QI v2.3 (Nonlinear Dynamics, Newcastle, UK). Metabolites were identified using progenesis QI (Waters Corporation, Milford, MA, USA) Data Processing Software, based on public databases (http://www.hmdb.ca/ (accessed on 20 September 2019); http://www.lipidmaps.org/ (accessed on 20 September 2019)). The positive and negative data were combined and imported into the R ropls package. Principle component analysis (PCA) was carried out to visualize the metabolic alterations among experimental groups. Differentially accumulated metabolites (DAMs) were identified according to the following criteria: VIP > 1 and *p* < 0.05. The enrichment analysis of KEGG pathways was performed using R (version 4.0.4). The metabolome sequencing and analysis were conducted by Shanghai OE Biotech Co., Ltd, Shanghai, China.

### 4.6. Correlation Analysis of Transcript and Metabolite Profiles

The random forest method was performed to obtain biomarkers that distinguish the current group using sklearn software (version 0.24.1). Pearson correlation tests were used to explore the correlations between the DEGs and DAMs using pheatmap software (version 1.0.12). The differential genes and metabolites were mapped to the KEGG pathway simultaneously in the transcriptome and metabolome analyses. 

### 4.7. Quantitative Real-Time PCR Analysis

To validate the RNA-seq data, 1 μg total RNA of each sample was extracted using Trizol (Invitrogen, Carlsbad, CA, USA) for preparation of the template for a qRT-PCR assay. After DNase digestion, the first strand cDNA was synthesized according to the manufacturer’s recommended procedures (Invitrogen, Carlsbad, CA, USA). Real-time PCR was then performed in a F7500 Fast system (Applied Biosystems, Foster City, CA, USA) with SYBR™ Green PCR Master Mix (RR036A, TAKARA Bio, USA). The PCR conditions and data analysis were conducted as described by Chen et al. [71]. Data were normalized using *HvGAPDH* and *HvActin* as endogenous references. Primer pairs were designed using primer 3 software (http://www.ncbi.nlm.nih.gov/tools/primer-blast/ (accessed on 22 May 2022)), and all the primer sequences were shown in Supplementary Table S3. The amplification efficiency of genes was evaluated by the LinRegPCR software(version 10). 

### 4.8. Chlorophyll and Sucrose Content 

Chlorophyll content was measured using a chlorophyll meter (SPAD-502; Minolta, Tokyo, Japan). Sucrose content was measured and calculated according to the plant tissue sucrose content detection kit (Ke Ming, Su Zhou, China). Approximately 0.1–0.2 g leaf tissue was weighed, ground at room temperature, and extracted with 0.5 mL of extract buffer. After proper grinding to a powder, the sample was quickly transferred to a centrifuge tube and incubated in a water bath at 80 °C for 10 min, with shaking to mix the contents 3–5 times during the incubation. After cooling, the sample was centrifuged at 4000× *g* for 10 min at room temperature. Then, the supernatant was taken, and 2 mg destaining solution was added before further incubation at 80 °C for 30 min. After incubation, 0.5 mL extract solution was mixed again, cooled at room temperature and centrifuged at 4000× *g* for 10 min at room temperature. The final supernatant was taken for analysis, and the absorbance of the mixture was recorded at 480 nm. Ten independent biological replicates were analyzed for all treatments included.

### 4.9. Statistical Data Analysis

Unless specified otherwise, comparisons among different treatments were tested by the least significant difference (LSD) test using SPSS statistical software(version 21.0). The difference at 0.05 level (*p* < 0.05) was considered to be significant.

## 5. Conclusions

A homozygous barley DH line (named DH20), which was produced by mutagenesis combined with microspore technologies, exhibited better performance on fresh shoot weight, dry shoot weight, K^+^/Na^+^ ratio and photosynthetic characteristics than the control under salt stress conditions. Transcriptome and metabolome analyses revealed nine biomarkers and five significantly enriched pathways that could contribute to the salt tolerance in DH20. Taken together, these results suggest that DH20 may enhance salt tolerance by promoting lipid metabolism, maintaining energy metabolism and decreasing amino acid metabolism. Our results suggest that the combination of mutagenesis with microspore culture technology is an efficient approach to rapidly generate DH lines for the improvement of salt tolerance.

## Data Availability

Data have been deposited with the National Center for Biotechnology Information under Submission ID: SUB13751180 and Bio Project ID: PRJNA1004076.

## Supplementary Materials

The supporting information can be downloaded at: https://www.mdpi.com/article/10.3390/ijms242316757/s1.
