# Supplementary material for: Transcriptomic and Metabolomic Analyses Reveal the Importance of Lipid Metabolism and Photosynthesis Regulation in High Salinity Tolerance in Barley (Hordeum vulgare L.) Leaves Derived from Mutagenesis Combined with Microspore Culture
Source: Int J Mol Sci. 2023 Nov 25;24(23):16757. doi: 10.3390/ijms242316757 (PMC10705989; doi:10.3390/ijms242316757)
Supplement: Supplementary file 1 [file ijms-24-16757-s001.zip › ijms-2725229-supplementary.pdf]

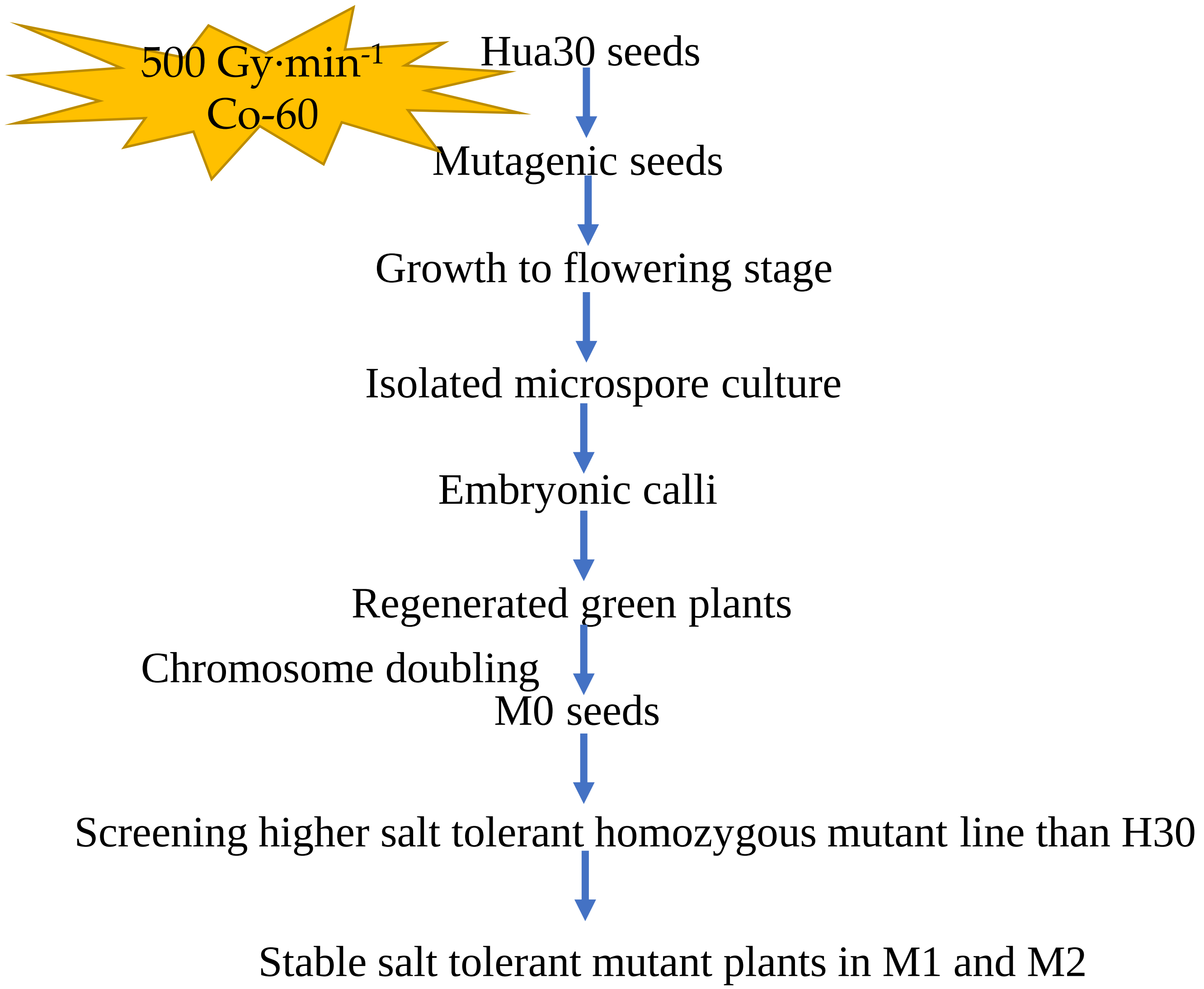

Figure S1. Breeding scheme for the production of salt tolerant homozygous mutant plants

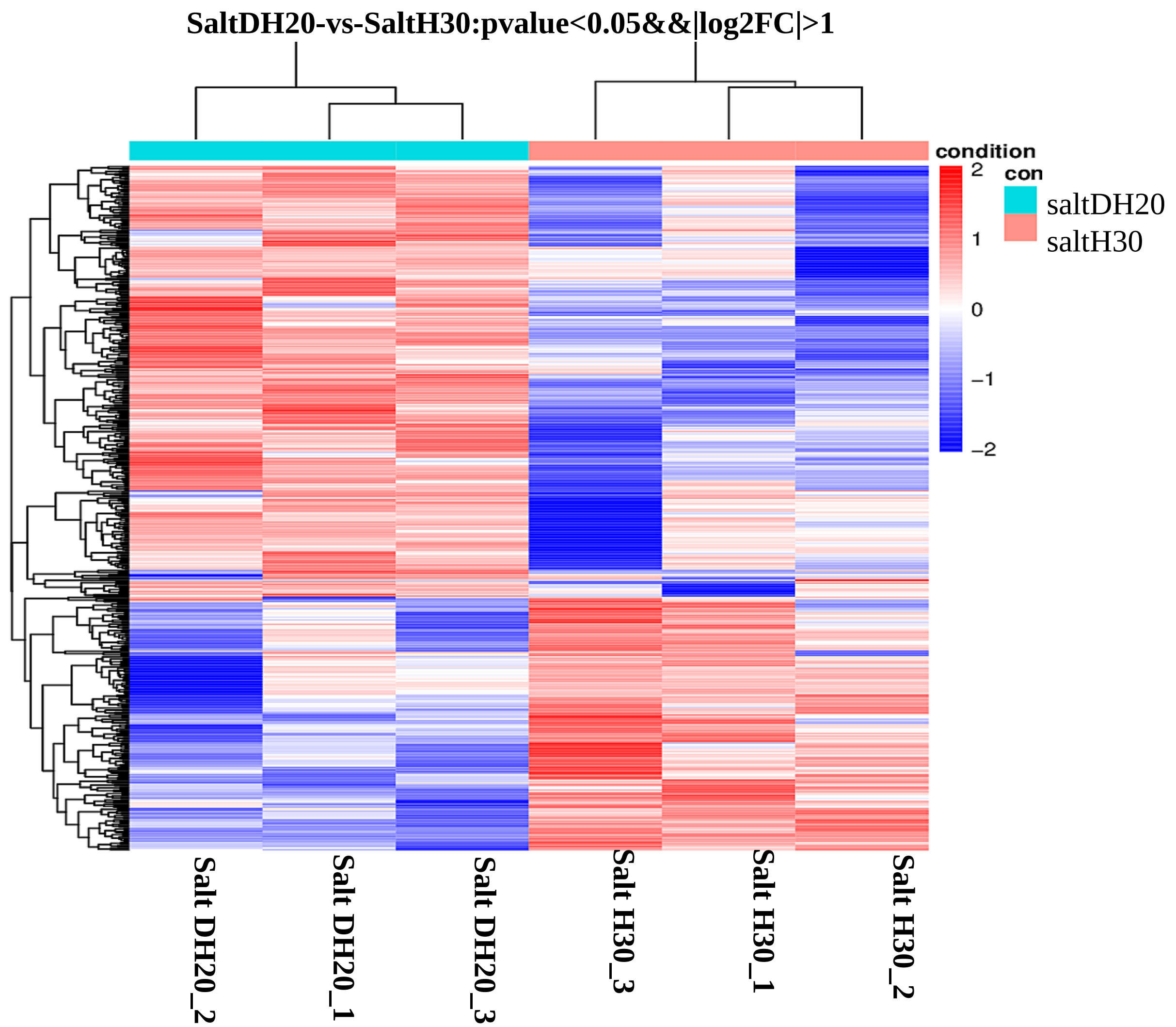

Figure S2. Heatmap analysis of specifically 462 DEGs. Samples and biological replicates of DH20 and H30 are shown. The color gradient (upper right) indicates the pattern of expression from lowly (blue)to highly (red) expressed genes and ranges from −2 to +2 fold changes in expression.

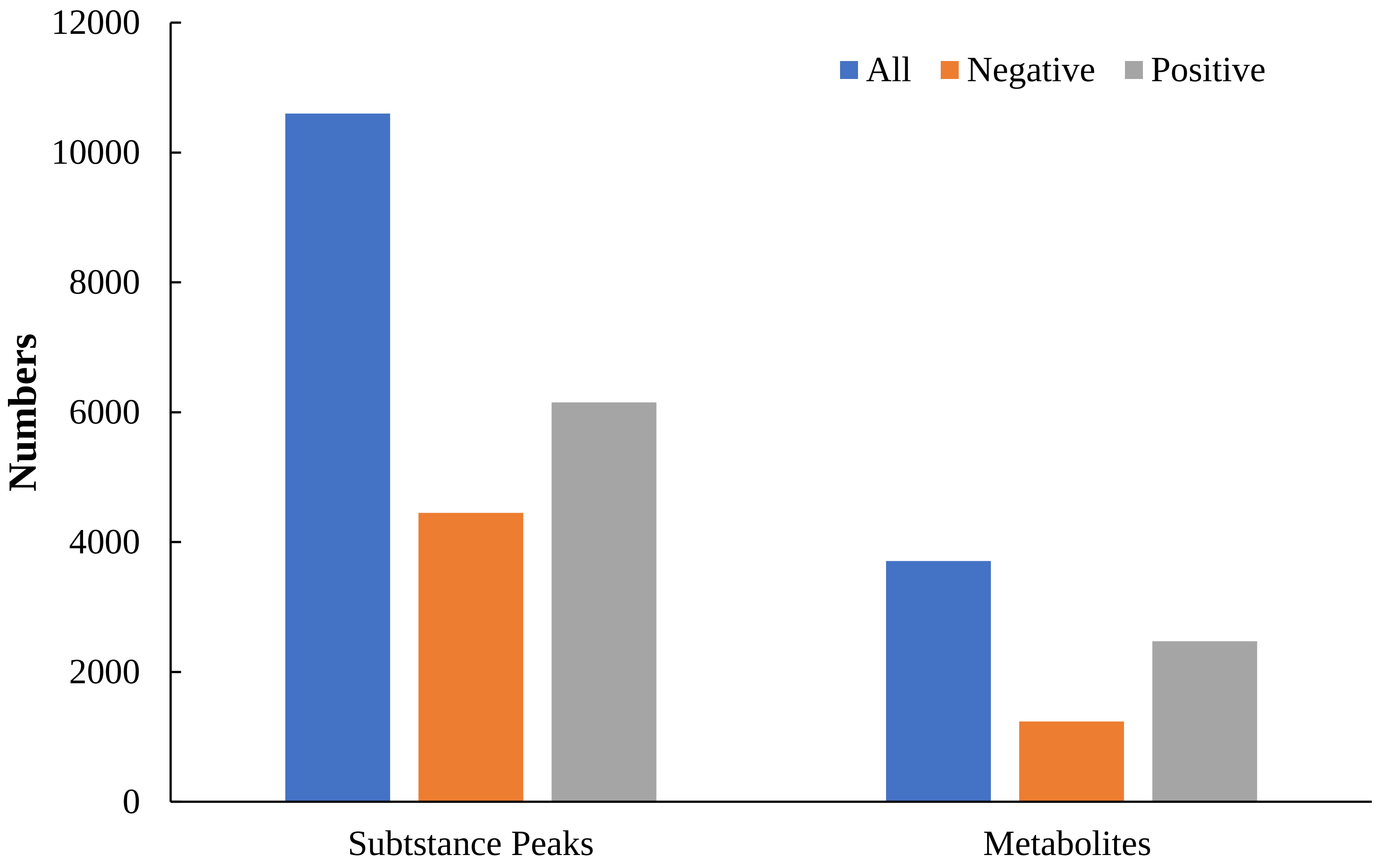

Figure S3. Statistical of substance peaks and metabolites

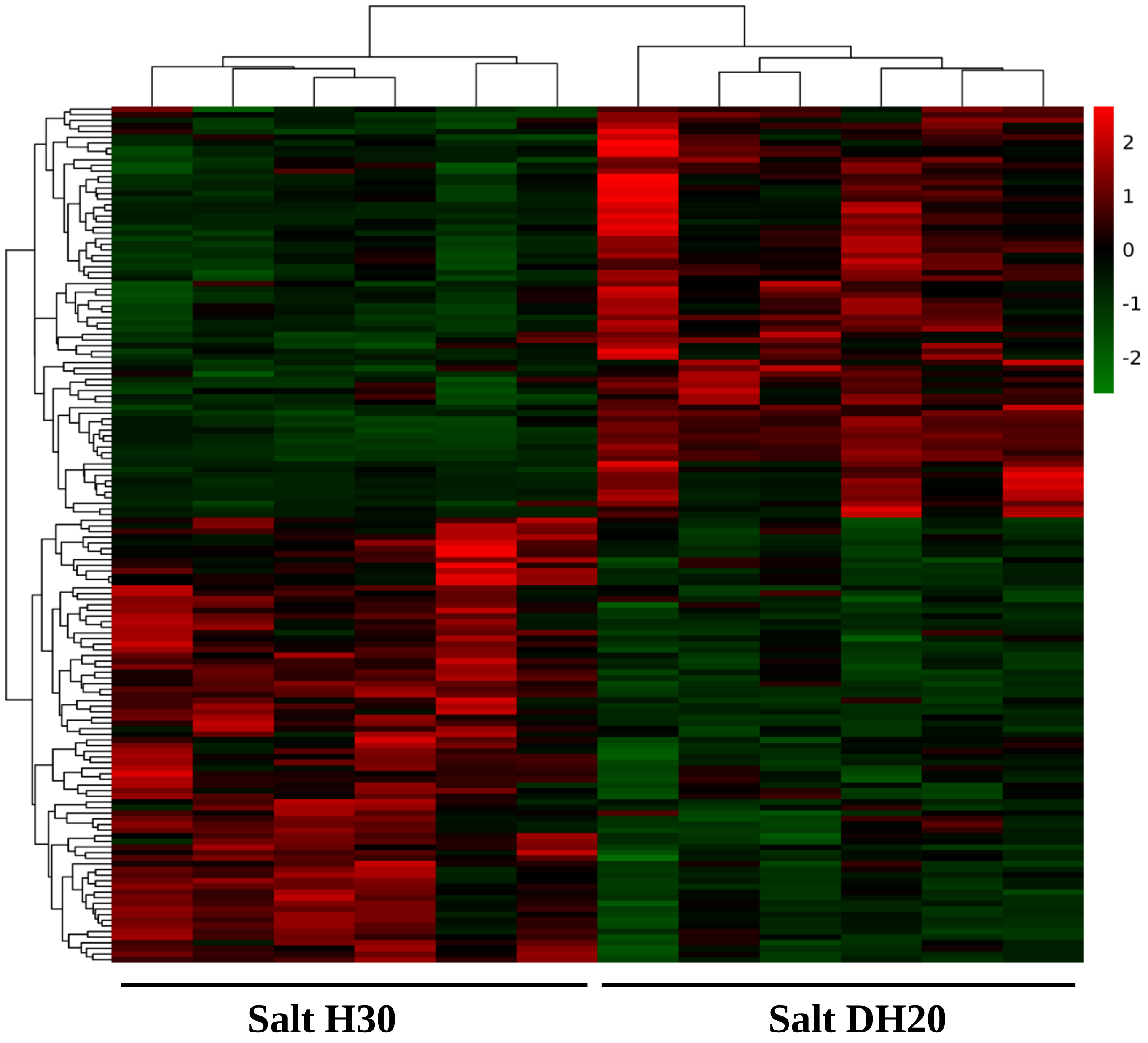

Figure S4. Heatmap analysis of 152 DAMs

Table S1. Leaf photosynthetic properties in H30 and DH20 under control and salt conditions

	LSP ($\mu mol\ m^{-2}\ s^{-1}$)	AQE ($\mu mol\ m^{-2}\ s^{-1}$)	Amax ($\mu mol\ m^{-2}\ s^{-1}$)	Vcmax ($\mu mol\ m^{-2}\ s^{-1}$)	Jmax ($\mu mol\ m^{-2}\ s^{-1}$)	Fv’/Fm’
CKH30	648±120.4b	0.03±0.002a	5.98±1.196b	16.83±2.61a	12.06±2.41a	0.52±0.01a
CKDH20	884±185.60a	0.04±0.006a	10.20±2.01a	10.83±2.05b	7.91±1.58b	0.51±0.01a
Salt H30	216±38.88B	0.007±0.0014A	0.95±0.19B	1.32±0.26B	0.74±0.15B	0.34±0.06B
SaltDH20	256±43.52A	0.007±0.00105A	1.25±0.26A	1.55±0.31A	1.32±0.26A	0.46±0.03A

LSP, light saturation point; AQE, apparent quantum efficiency; Amax, maximum net photosynthetic rate; Vcmax, maximum rate of RuBP carboxylation; Jmax, maximum rates of electron transport; Fv’/Fm’, the maximal photochemical efficiency of PSII.

Table S2. Summary of quality control for RNA-seq data

Sample	Raw_reads	Raw_bases	Clean_reads	Clean_bases	Valid_bases	Q30	GC
CK DH20_1	69.67M	10.45G	61.17M	8.81G	84.26%	90.46%	56.59%
CK DH20_2	64.59M	9.69G	57.34M	8.46G	87.28%	90.87%	56.98%
CK DH20_3	69.37M	10.41G	57.08M	8.10G	77.85%	87.61%	56.29%
CK H30_1	71.97M	10.80G	54.67M	7.74G	71.69%	84.22%	57.41%
CK H30_2	67.21M	10.08G	56.73M	8.36G	82.91%	88.57%	56.32%
CK H30_3	46.54M	6.98G	41.49M	6.09G	87.20%	89.87%	56.48%
Salt DH20_1	65.79M	9.87G	63.78M	9.12G	92.45%	95.04%	55.29%
Salt DH20_2	85.30M	12.79G	49.79M	7.10G	55.48%	74.46%	57.34%
Salt DH20_3	61.69M	9.25G	59.76M	8.61G	93.06%	94.94%	55.84%
Salt H30_1	69.30M	10.39G	61.69M	8.88G	85.45%	91.06%	55.92%
Salt H30_2	68.00M	10.20G	54.43M	7.89G	77.32%	85.13%	56.23%
Salt H30_3	63.82M	9.57G	62.35M	8.86G	92.51%	95.66%	54.94%

Table S3. Primer information for qRT-PCR

Gene	primer sequence (5'-3')	primer sequence (3'-5')
HORVU1Hr1G067480	TGTGCCCCATACTCAAAACA	GTATCGCCTCAAGGATGGAA
HORVU3Hr1G056830	AGGTGTACGACCACCTCTGG	GCCCTACCTCAACCATCTCA
HORVU1Hr1G071890	GAATCTGGCCTTTTGGATGA	TTGGTCAATGCAACACAGGT
HORVU7Hr1G050660	AGTACCTGGGAGGGATGGAG	TGGTTTCATGAGCTGGTACG
HORVU4Hr1G006850	TGCATGGTGACTGGTGATCT	ACTCTTGACGAGGACGCAGT
HORVU1Hr1G093460	CTACACCATCCCCGAAGAGA	ACCAAACCAGCATTCACCTC
HORVU2Hr1G115960	GCGTTCTGCATGAATCTTGA	TGCTTGGTATTGTTGGGTGA
HORVU5Hr1G117910	ACCGTCGGCTTATATCAACG	GAACACAAGGGGTCATGCTT
HORVU4Hr1G011160	CGTTAGCAGGCATCAGTCAA	TTCTTTCCAAAACCCACGAC
HORVU5Hr1G095060	CCTCTTCGTGGACAAGGTGT	CCTGCCATGGTCATCTACCT
HORVU1Hr1G074460	CAATGGTTGACACCGAACTG	CTGGGATTTCAGCACCAACT
HORVU7Hr1G007220	TGACTGGTCTGGTTGAGCTG	CTGCTCCTCCTTGTCCTTTG
